# Exergetic Analysis of a Cryogenic Air Separation Unit

**DOI:** 10.3390/e24020272

**Published:** 2022-02-13

**Authors:** Sorin Bucsa, Alexandru Serban, Mugur C. Balan, Claudia Ionita, Gabriel Nastase, Catalina Dobre, Alexandru Dobrovicescu

**Affiliations:** 1 Department of Engineering Thermodynamics, University Politehnica of Bucharest, 060042 Bucharest, Romania; sobucsa@yahoo.com (S.B.); alexandru.serban@upb.ro (A.S.); claudia.ionita@upb.ro (C.I.); traznasa@gmail.com (G.N.); 2 Department of Thermodynamics, Technical University of Cluj-Napoca, 400114 Cluj-Napoca, Romania; mugur.balan@termo.utcluj.ro

**Keywords:** chemical exergy, rectification column, compression unit

## Abstract

This case study analyzes a cryogenic air separation unit (ASU) with a production of ${\dot{V}}_{O_{2}}=58,300\;\left[\frac{m^{3}N}{h}\right]$ of gaseous oxygen with a concentration greater than 98.5%, operating in Romania on a steel plant platform. The goal of the paper is to provide an extensive model of exergetic analysis that could be used in an optimization procedure when decisional parameters are changed or structural design modifications are implemented. For each key part of the Air Separation Unit, an exergetic product and fuel were defined and, based on their definition, the coefficient of performance of each functional zone was calculated. The information about the magnitude of the exergetic losses offers solutions for their future recovery. The analysis of the exergy destructions suggests when it is worth making a larger investment. The exergetic analysis of the compression area of the ASU points out an exergy destruction and loss of 37% from the total plant’s electrical energy input. The exergy loss with the heat transferred to the cooling system of compressors can be recovered; for the exergy destruction portion, the challenge between investment and operating costs should be considered. The exergy destruction of the air separation columns found the High Pressure Column (HPC) to be more destructive than the Low Pressure Column. The share of the exergy destruction in the total plant’s electrical energy input is 8.3% for the HPC. The local COP of the HPC, calculated depending on the total exergy of the local product and fuel, is 62.66%. The calculus of the air separation column is performed with the ChemSep simulator.

## 1. Introduction

On a large scale, the cost of separating oxygen and other air gases, such as nitrogen, argon, krypton, and neon, is far cheaper and the purity of the products is far higher when using the cryogenic distillation route.

A growing application for oxygen today is for dealing with polluted rivers which have been deoxygenated.

Nitrogen is the other main component of the air. Today, the overall demand for nitrogen gas exceeds that for oxygen. Since nitrogen is inert and non-toxic, it has found many uses ranging from food preservation to the blanketing of chemical engineering and other industrial processes. In particular, the semiconductor industry uses large quantities; its demand for higher and still higher purity nitrogen arises from the increasing miniaturization and complexity of the integrated circuit chips being manufactured. These ultra-high levels of purity can be easily achieved by cryogenic distillation of air.

Argon is, chemically, totally inert, even at high temperatures. The demand for argon, as a shield gas, in arc-welding and in the high technology metallurgical industry has grown even more than the demand for nitrogen.

The products of the industrial gas industry have also become featured in biological and medical advances. Oxygen is well known for its life-supporting role in hospitals.

Cryogenic Air Separation Units (CASU) are large electrical energy consumers. In the general concern of how to make such systems more efficient, the thermodynamic approach plays a crucial role when estimating the performance of any functional zone. A close-to-realistic assessment of the product and fuel of every operating area provides the chance to identify opportunities for optimization.

An analysis based on the first and second principles of thermodynamics and the exergy concept provides the instrument for pointing out the location and magnitude of malfunctions associated with the operating processes.

The production of pure oxygen efficiently and at industrial scale is a topic of great interest.

Ebrahim et al. [1] recognizes that the most economical method of producing large quantities of oxygen and nitrogen is by cryogenic air separation. Authors focus their analysis on a two column ASU and use as an investigation instrument the exergetic analysis combined with economics. The exergoeconomic analysis reveals that in order to provide profit, such a system should have a larger production than 9 kg/s of O_2_.

Ghorbani et al. [2] conducted an exergetic and exergoeconomic analysis of a natural gas separation installation, calculating the exergy destruction and loss in the key parts of the system. In terms of thermoeconomic costs, they found the compression area to be the most expensive.

Tesch et al. [3] integrated a natural gas liquefaction system with a cryogenic air separation system. The analysis of the benefits of such a combined system is based on an advanced exergy study. The introduction of the natural gas liquefaction increases the overall exergetic efficiency of the ASU alone by 10%.

Konoghe [4] undertook an exergetic analysis of a natural gas liquefaction unit operating in cascades. The author developed relationships for calculating the exergy destruction in the key parts of the system. The exergetic analysis indicates a great potential for improvements. The paper offers a correlation between the properties of the incoming and outgoing natural gas streams that indicates the minimum work input for liquefaction.

The subject of integrating a liquefying natural gas with an ASU has also been analyzed by Chen et al. [5]. Based on the first and second laws of thermodynamics, a comparative analysis involving different types of rectification columns in the ASU was performed. The coupling of the ASU with the LNG system improved the overall performance of the composed system.

Acikkalp et al. [6] realized the case study of an ASU based on energy and exergy analysis. Two indicators were used—the energetic improvement potential rate (EIP) and the exergetic improvement potential rate (ExIP). The plant was found to reach an energetic efficiency of 45% and an exergetic efficiency of 13%.

Tesch, Morosuk and Tsatsaronis [7] performed an advanced exergy analysis of the regasification of the liquid natural gas as a part of a more complex ASU. Options for new designs were suggested by the results given by the exergetic analysis.

Rong at al. [8] analyzed an air separation unit in an attempt to recover as much as possible from the exergy loss, with the heat transferred to the cooling system of the compressor’s zone. A part of this potential will run an organic Rankine cycle. the rest being used for the dehumidification area of the ASU.

Banaszkiewicz [9] suggested recovering the exergy expended for the liquefaction of LNG, lost in the regasification process, for the separation of oxygen from the air in an adsorption system. The latent heat of vaporization of the LNG was used for cooling the adsorption bed and increasing, in this way, its adsorptive capacity.

The above review reveals the general understanding that only a method based on the exergetic analysis [10] offers major insights into the true operation of the system that will enable decisions for improvement.

The present work succeeds in deeply analyzing each technological process. The novelty represents the splitting of total exergy into its thermo-mechanical and chemical components [11,12,13] that aids in analyzing the formation of the local product and fuel offerings. In addition, variants of estimating the performance of each operating area are reviewed.

Estimating the local COP as close to reality as possible represents the major opportunity in the search for an optimum operating regime or constructive solution [14].

The intention of this article is not to critically analyze and compare different technologies of gas mixture separation. The main goal of the paper is to provide a logical and explicit exergy analysis model that could be applied in any strategy of structural and functional optimization of a cryogenic air separation unit.

## 2. Installation Description. Functional Scheme

**Figure 1:**
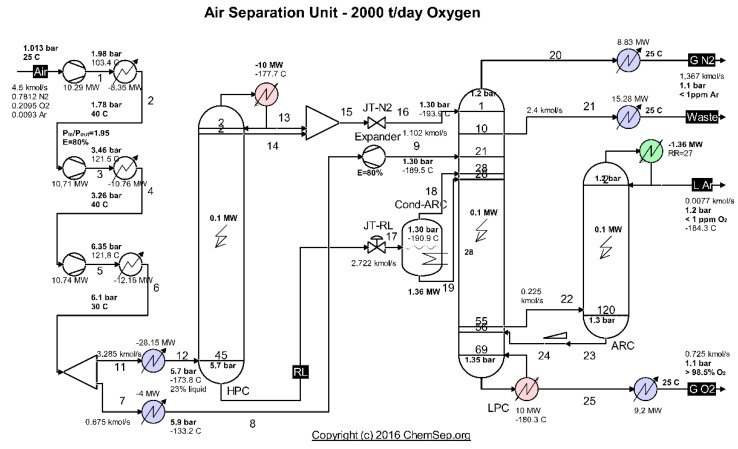
Diagram of the cryogenic air separation installation. Source: Harry Kooijman (2006) chemsep.org.

The case study analyzes a cryogenic air separation unit (ASU) with a production of ${\dot{V}}_{O_{2}}=58,300\;\left[\frac{m^{3}N}{h}\right]$ of gaseous oxygen with a concentration greater than 98.5%, operating in Romania on a steel plant platform. The scheme of the installation is presented in Figure 1.

The separation of the air occurs in two distillation columns that operate at different pressures, coupled by a heat exchanger acting as a condenser for the high-pressure column (HPC) and evaporator (reboiler) for the low-pressure column (LPC). HPC operates at a pressure of about 6 bar and LPC of about 1.4 bar.

The compressed, dry and purified atmospheric air is cooled in the main recuperative heat exchanger close to its liquefaction temperature (−170 °C) and introduced into the high-pressure column (HPC).

In the high-pressure column (HPC), the air is separated by distillation into a stream of nitrogen gas, which accumulates at the top of the column, and a stream of liquid enriched in oxygen (about 30% O_2_) at the bottom.

The separation process is repeated in the low-pressure column (LPC).

The nitrogen gas in the high-pressure (and temperature) column (HPC) is partially condensed in the vaporizer condenser that couples the two columns by transferring heat to the liquid enriched in oxygen in the low-pressure column (LPC) which vaporizes at about −180 °C.

Part of the liquid nitrogen at the top of the high-pressure column ensures the reflux current and the remaining N_2_ liquefy together with the nitrogen gas and are throttled in a throttling valve to the pressure of the low-pressure column, feeding in the LPC on the top at the liquid state.

From the HPC base, the liquid enriched in O_2_ is throttled and introduced in the middle of the low-pressure column where the distillation process is continued.

Distillation products (separate gases) are discharged from the low-pressure column.

To supplement the cold capacity of the installation, a fraction of about 15% of the compressed air is expanded into an expander before being introduced into the low-pressure column.

The simulation of the operation of air separation columns was performed using the ChemSep simulator [15].

The modeling of the operating of the ASU is based on the exergetic analysis.

## 3. Exergetic Analysis

**Figure 2:**
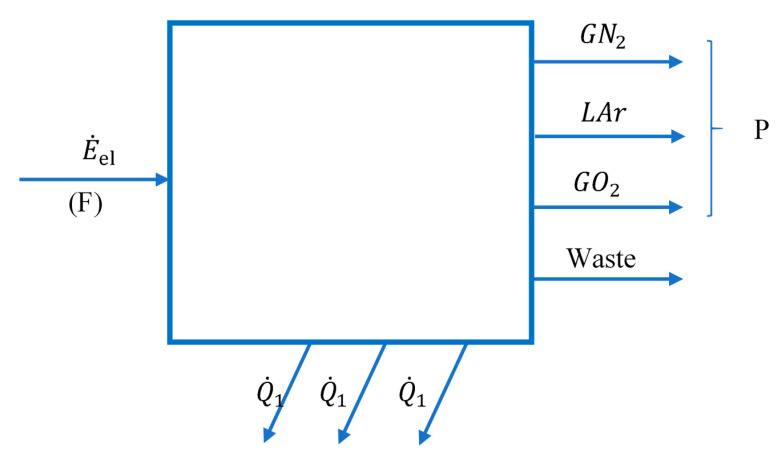
Diagram of interactions with the external environment of the global system of the cryogenic air separation plant.

Looking at the installation as a whole (Figure 1), products, exergetic fuel and global system losses can be identified (Figure 2).

The exergetic performance coefficient of the whole system is:

(1) $$\;COP_{ex}=\frac{P}{F}=\frac{\dot{E}x_{GN_{2}}^{TOT}+\dot{E}x_{LAr}^{TOT}+\dot{E}x_{GO_{2}}^{TOT}}{{\dot{E}}_{el}}\;$$

Since the compression zone is provided by a three-stage compression compressor:

(2) $${\dot{E}}_{el}={\dot{W}}_{1}+{\dot{W}}_{2}+{\dot{W}}_{3}\;$$

The terms of the product of the installation are calculated as follows:

(3) $$\dot{E}x_{GN_{2}}^{TOT}=\dot{E}x_{GN_{2}}^{TM}+\dot{E}x_{GN_{2}}^{CH}\;$$

(4) $$\dot{E}x_{GN_{2}}^{TM}=n_{GN_{2}}\left\{{\bar{h}}_{N_{2}}\left(T,\;p\right)-{\bar{h}}_{N_{2}}\left(T_{0},\;p_{0}\right)-T_{0}\left[{\bar{s}}_{N_{2}}\left(T,\;p\right)-{\bar{s}}_{N_{2}}\left(T_{0},\;p_{0}\right)\right]\right\}\;$$

(5) $$\dot{E}x_{GN_{2}}^{CH}=n_{GN_{2}}\cdot \bar{R}\cdot T_{0}\cdot ln\frac{{\bar{x}}_{N_{2}}}{{\bar{x}}_{N_{2}}^{0}}\;$$

The reference state is characterized by $t_{0}=25$ °C, $p_{0}=1.013\;\mathrm{bar}$ and air composition ${\bar{x}}_{N_{2}}=0.7812$; ${\bar{x}}_{O_{2}}=0.2095$; ${\bar{x}}_{Ar}=0.0093$.

(6) $$\dot{E}x_{GO_{2}}^{TOT}=\dot{E}x_{GO_{2}}^{TM}+\dot{E}x_{GO_{2}}^{CH}\;$$

(7) $$\dot{E}x_{GO_{2}}^{TM}=n_{GO_{2}}\left\{{\bar{h}}_{O_{2}}\left(T,\;p\right)-{\bar{h}}_{O_{2}}\left(T_{0},\;p_{0}\right)-T_{0}\left[{\bar{s}}_{O_{2}}\left(T,\;p\right)-{\bar{s}}_{O_{2}}\left(T_{0},\;p_{0}\right)\right]\right\}\;$$

(8) $$\dot{E}x_{GO_{2}}^{CH}=n_{GO_{2}}\cdot \bar{R}\cdot T_{0}\cdot ln\frac{{\bar{x}}_{O_{2}}}{{\bar{x}}_{O_{2}}^{0}}\;$$

(9) $$\dot{E}x_{LAr}^{TOT}=\dot{E}x_{LAr}^{TM}+\dot{E}x_{LAr}^{CH}\;$$

(10) $$\dot{E}x_{LAr}^{TM}=n_{LAr}\left\{{\bar{h}}_{Ar}\left(T,\;p\right)-{\bar{h}}_{Ar}\left(T_{0},\;p_{0}\right)-T_{0}\left[{\bar{s}}_{Ar}\left(T,\;p\right)-{\bar{s}}_{Ar}\left(T_{0},\;p_{0}\right)\right]\right\}\;$$

(11) $$\dot{E}x_{LAr}^{CH}=n_{LAr}\cdot \bar{R}\cdot T_{0}\cdot ln\frac{{\bar{x}}_{Ar}}{{\bar{x}}_{Ar}^{0}}\;$$

The values of the fuels and products of the global system are presented in the Table 1 and Table 2.

The value of the exergetic performance coefficient (Equation (1)) is $COP_{ex}=0.14$.

The exergetic balance equation of the global installation is:

(12) F=P+L+I 

in which *L* represents losses of exergy thrown into the external environment and *I* destruction (consumption) of exergy caused by internal irreversibilities.

From the exergetic balance Equation (12) it follows that

(13) $$COP_{ex}=\frac{P}{F}=1-\frac{L+I}{F}\;$$

It further follows that the efficiency of the installation can be achieved by reducing losses and destruction of exergy from fuel consumption.

It is noted that

(14) $$\psi_{j}=\frac{{\left(L+I\right)}_{j}}{F}\;$$

as the share in fuel consumption of the loss and destruction of exergy in a functional area.

The total exergy current of the gaseous nitrogen discharged in the form of WASTE (Figure 1 and Figure 2), together with the exergies of the heat flows discharged to the outside through the cooling system of the compressors, represents losses for the global system, losses which we will try to recover during the optimization procedure.

The share of this total loss in the total fuel consumption of the global installation is:

(15) $$\psi_{L}=\frac{\dot{E}x_{Waste}^{TOT}+\left|\dot{E}x_{Q_{1}}\right|+\left|\dot{E}x_{Q_{2}}\right|+\left|\dot{E}x_{Q_{3}}\right|}{\left|{\dot{W}}_{1}\right|+\left|{\dot{W}}_{2}\right|+\left|{\dot{W}}_{3}\right|}\cdot 100\;$$

**Figure 3:**
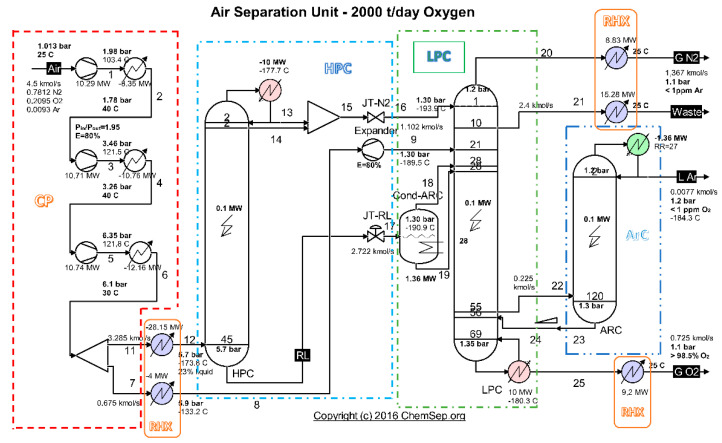
Scheme of the cryogenic air separation installation—functional areas. Source: Harry Kooijman (2006) chemsep.org.

To identify the place where losses and destructions take place, the installation is divided into functional areas (Figure 3).

### 3.1. Compression Area

**Figure 4:**
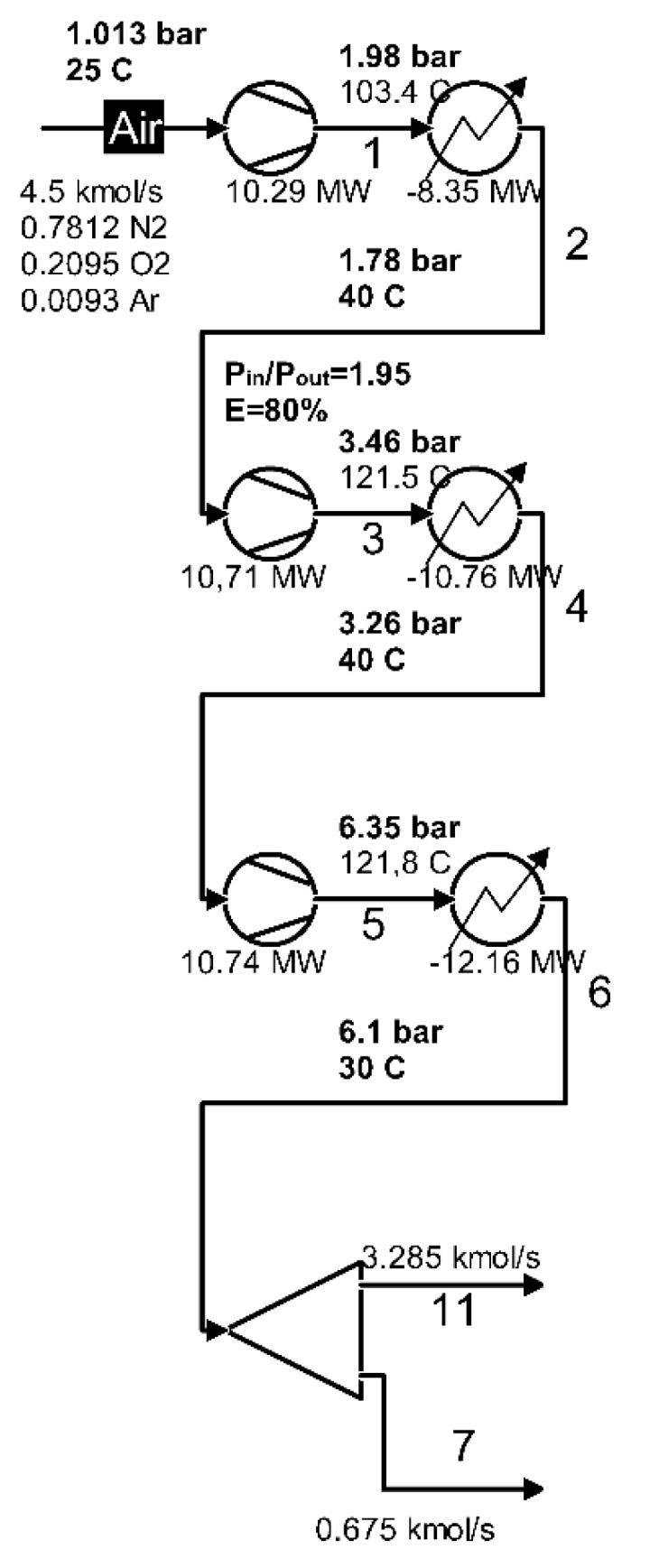
Compression zone of the air separation installation.

The compression zone as part of the cryogenic air separation installation is shown in Figure 4.

The exergetic balance equation of the compression zone is:

(16) $$\sum^{\;}\dot{E}x_{i}=\sum^{\;}\dot{E}x_{e}+\dot{\dot{I}\;}$$

In the energy balance Equation (16), both the input and output energies are in absolute value.

(17) $$\dot{E}x_{AIR}+\left|{\dot{W}}_{1}\right|+\left|{\dot{W}}_{2}\right|+\left|{\dot{W}}_{3}\right|=\dot{E}x_{11}+\dot{E}x_{7}+\left|\dot{E}x_{Q_{1}}\right|+\left|\dot{E}x_{Q_{2}}\right|+\left|\dot{E}x_{Q_{3}}\right|+\dot{I}\;$$

Equation (17) written with an economic connotation becomes:

(18) $$F_{cp}=P_{cp}+{\left(L+I\right)}_{cp}\;$$

**Figure 5:**
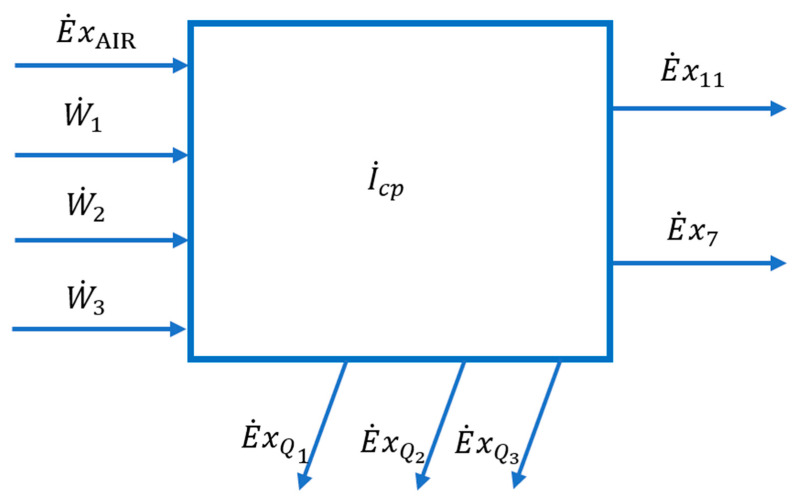
Scheme of interactions with the external environment of the compression zone of the cryogenic air separation installation.

The scheme of the interactions of the compression zone with the outside is presented in Figure 5.

Where *F* is the fuel of the area equal to the fuel of the overall system

(19) $$F_{cp}=F=\left|{\dot{W}}_{1}\right|+\left|{\dot{W}}_{2}\right|+\left|{\dot{W}}_{3}\right|\;$$

and *P* is the product of the area and represents its net exergy increase.

(20) $$P_{cp}=\dot{E}x_{11}+\dot{E}x_{7}-\dot{E}x_{AIR}\;$$

(21) $$L_{cp}=\left|\dot{E}x_{Q_{1}}\right|+\left|\dot{E}x_{Q_{2}}\right|+\left|\dot{E}x_{Q_{3}}\right|\;$$

has the significance of the loss of energy, with the heat evacuated to the outside in the intermediate coolers and the final cooler of the compression zone.

*İ* is the destruction of exergy in the compression process.

Equation (18) becomes:

(22) $$\left|{\dot{W}}_{1}\right|+\left|{\dot{W}}_{2}\right|+\left|{\dot{W}}_{3}\right|=\left(\dot{E}x_{11}+\dot{E}x_{7}-\dot{E}x_{AIR}\right)+\left|\dot{E}x_{Q_{1}}\right|+\left|\dot{E}x_{Q_{2}}\right|+\left|\dot{E}x_{Q_{3}}\right|+\dot{\dot{I}\;}$$

Exergetic quantities are calculated as follows:

(23) $$\dot{E}x_{11}=n_{11}\left\{{\bar{h}}_{11}\left(T_{11},\;p_{11}\right)-{\bar{h}}_{11}\left(T_{0},\;p_{0}\right)-T_{0}\left[{\bar{s}}_{11}\left(T_{11},\;p_{11}\right)-{\bar{s}}_{11}\left(T_{0},\;p_{0}\right)\right]\right\}\;$$

(24) $$\dot{E}x_{7}=n_{7}\left\{{\bar{h}}_{7}\left(T_{7},\;p_{7}\right)-{\bar{h}}_{7}\left(T_{0},\;p_{0}\right)-T_{0}\left[{\bar{s}}_{7}\left(T_{7},\;p_{7}\right)-{\bar{s}}_{7}\left(T_{0},\;p_{0}\right)\right]\right\}\;$$

(25) $$\dot{E}x_{AIR}=n_{AIr}\left\{{\bar{h}}_{AIR}\left(T_{AIR},\;p_{AIR}\right)-{\bar{h}}_{AIR}\left(T_{0},\;p_{0}\right)-T_{0}\left[{\bar{s}}_{AIR}\left(T_{AIR},\;p_{AIR}\right)-{\bar{s}}_{AIR}\left(T_{0},\;p_{0}\right)\right]\right\}\;$$

(26) $$\left|\dot{E}x_{Q_{1}}\right|+{\dot{I}}_{cp_{1}}=n_{AIR}\left\{{\bar{h}}_{1}\left(T_{1},p_{1}\right)-{\bar{h}}_{2}\left(T_{2},p_{2}\right)-T_{0}\left[{\bar{s}}_{1}\left(T_{1},p_{1}\right)-{\bar{s}}_{2}\left(T_{2},p_{2}\right)\right]\right\}\;$$

(27) $$\left|\dot{E}x_{Q_{2}}\right|+{\dot{I}}_{cp_{2}}=n_{AIR}\left\{{\bar{h}}_{3}\left(T_{3},p_{3}\right)-{\bar{h}}_{4}\left(T_{4},p_{4}\right)-T_{0}\left[{\bar{s}}_{1}\left(T_{3},p_{3}\right)-{\bar{s}}_{4}\left(T_{4},p_{4}\right)\right]\right\}\;$$

(28) $$\left|\dot{E}x_{Q_{3}}\right|+{\dot{I}}_{cp_{3}}=n_{AIR}\left\{{\bar{h}}_{5}\left(T_{5},p_{5}\right)-{\bar{h}}_{6}\left(T_{6},p_{6}\right)-T_{0}\left[{\bar{s}}_{5}\left(T_{5},p_{5}\right)-{\bar{s}}_{6}\left(T_{6},p_{6}\right)\right]\right\}\;$$

The exergy destruction inside each compression stage due to the irreversibility of friction is calculated based on the Gouy-Stodola theorem [16] that accounts for the entropy generation that accompanies any irreversible process.

(29) $${\dot{I}}_{cp}={\dot{I}}_{cp_{1}}+{\dot{I}}_{cp_{2}}+{\dot{I}}_{cp_{3}}\;$$

(30) $${\dot{I}}_{cp_{1}}=n_{AIR}\cdot T_{0}\left({\bar{s}}_{1}-{\bar{s}}_{0}\right)\;$$

(31) $${\dot{I}}_{cp_{2}}=n_{AIR}\cdot T_{0}\left({\bar{s}}_{3}-{\bar{s}}_{2}\right)\;$$

(32) $${\dot{I}}_{cp_{3}}=n_{AIR}\cdot T_{0}\left({\bar{s}}_{6}-{\bar{s}}_{5}\right)\;$$

The share of exergy losses and damage in the total fuel consumption of the installation is:

(33) $$\psi_{cp}=\frac{{\left(L+I\right)}_{cp}}{{\left|\dot{W}\right|}_{1}+{\left|\dot{W}\right|}_{2}+{\left|\dot{W}\right|}_{3}}\;$$

### 3.2. High Pressure Distillation Column (HPC)

**Figure 6:**
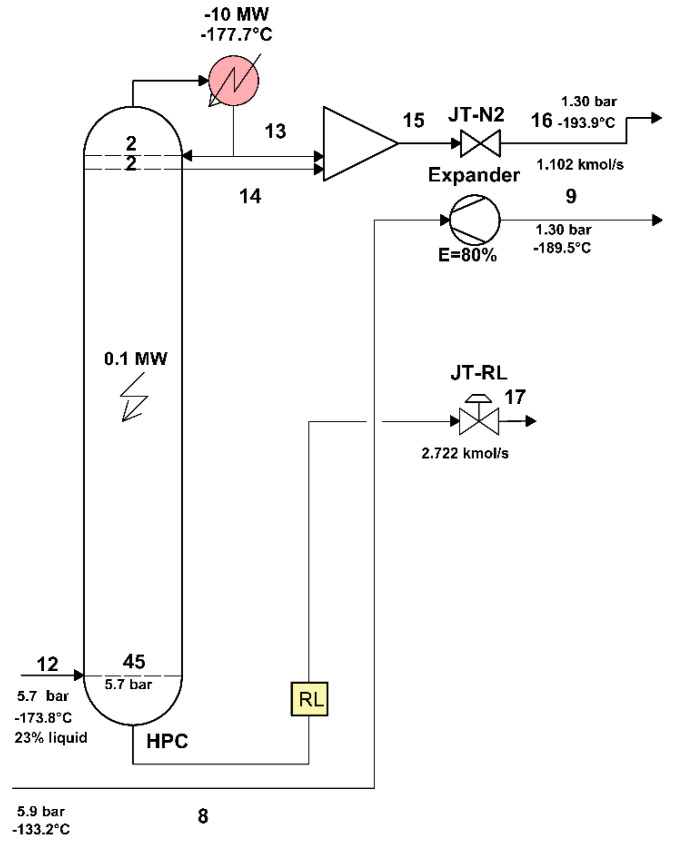
The area of the high-pressure distillation column of the air separation plant.

The area of the HPC distillation column, separated from the rest of the plant, is shown in Figure 6.

**Figure 7:**
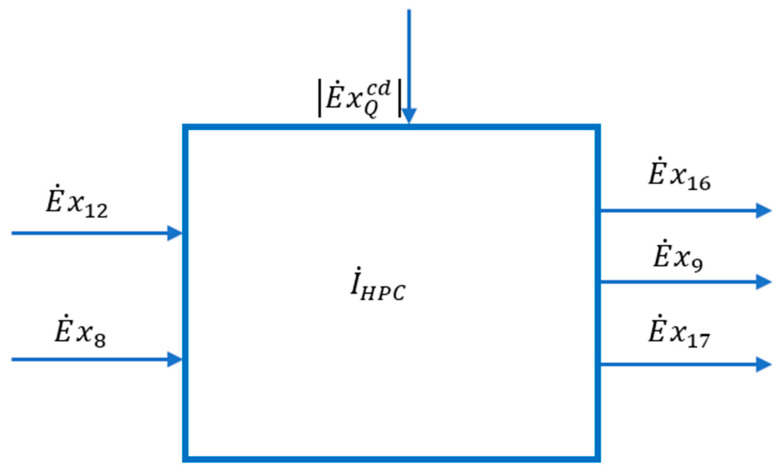
Scheme of interactions with the external environment of the high-pressure distillation column (HPC) area.

To specify the membership in the fuel (F) or product (P) of the heat discharged from the condenser of the high pressure and temperature column and transferred to the reboiler (evaporator) of the low-pressure column, write the exergetic balance equation of the HPC in its general algebraic form (in which each term contains its sign) (Figure 7).

(34) $$\sum^{\;}\dot{E}x_{Q}=\sum^{\;}\dot{E}x_{e}-\sum^{\;}\dot{E}x_{i}+\sum^{\;}\dot{W}+\dot{I}\;$$

The effect of heat penetration from the environment is neglected in a first phase and it is considered that the heat exchange with the outside in the HPC is performed only in the condenser. Equation (34) becomes:

(35) $$\dot{E}x_{Q}^{cd}=\dot{E}x_{16}+\dot{E}x_{9}+\dot{E}x_{17}-\dot{E}x_{12}-\dot{E}x_{8}+\dot{I}\;$$

The exergy of the condensing heat is:

(36) $$\dot{E}x_{Q}^{cd}=\dot{\dot{Q}\left(1-\frac{T_{0}}{T}\right)}\;$$

It is observed that:

The HPC column yields heat $\dot{Q}<0$, $\dot{Q}=-\left|\dot{Q}\right|$.

The scheme of the interactions of the HPC area with its exterior can be found in Figure 7.

Heat is released at a temperature level $T<T_{0},\;\left(1-\frac{T_{0}}{T}\right)<0$

In the end,

(37) $$\dot{E}x_{Q}^{cd}=-\left|\dot{Q}\right|\left(1-\frac{T_{0}}{T}\right)=\left|\dot{Q}\right|\left(\frac{T_{0}}{T}-1\right)=\left|\dot{E}x_{Q}^{cd}\right|\;$$

Substituting in the exergetic balance equation of the high-pressure column (35) and rearranging the terms results in:

(38) $$\left|\dot{E}x_{Q}^{cd}\right|+\dot{E}x_{12}+\dot{E}x_{8}=\dot{E}x_{16}+\dot{E}x_{9}+\dot{E}x_{17}+\dot{I}\;$$

We can consider that the product (purpose) of the distillation column is to change the chemical composition of the inlet streams. The product is therefore the increase in the chemical exergy of the input currents.

To highlight the chemical component of the exergy, the exergy of the substance currents is split into the thermo-mechanical (physical) and the chemical component.

The exergetic balance Equation (38) becomes:

(39) $${\left|\dot{E}x\right|}_{Q}^{cd}+\dot{E}x_{12}^{TM}+\dot{E}x_{12}^{CH}+\dot{E}x_{8}^{TM}+\dot{E}x_{8}^{CH}=\dot{E}x_{16}^{TM}+\dot{E}x_{16}^{CH}+\dot{E}x_{9}^{TM}+\dot{E}x_{9}^{CH}+\dot{E}x_{17}^{TM}+\dot{E}x_{17}^{CH}+\dot{I}\;$$

The product is to increase the chemical exergy on the inlet-outlet path of the distillation column.

(40) $$P_{HPC}^{CH}=\dot{E}x_{16}^{CH}+\dot{E}x_{9}^{CH}+\dot{E}x_{17}^{CH}-\dot{E}x_{12}^{CH}-\dot{E}x_{8}^{CH}\;$$

Currents 8 and 9 are not processed in the distillation column, so that $\dot{E}x_{9}^{CH}=\dot{E}x_{8}^{CH}$.

Conseqently:

(41) $$P_{HPC}^{CH}=\dot{E}x_{16}^{CH}+\dot{E}x_{17}^{CH}-\dot{E}x_{12}^{CH}\;$$

(42) $$F_{HPC}^{CH}={\left|\dot{E}x\right|}_{Q}^{cd}+\dot{E}x_{12}^{TM}+\dot{E}x_{8}^{TM}-\dot{E}x_{16}^{TM}-\dot{E}x_{9}^{TM}-\dot{E}x_{17}^{TM}\;$$

(43) $$COP_{ex\_CH}^{HPC}=\frac{P_{HPC}^{CH}}{F_{HPC}^{CH}}=\frac{\dot{E}x_{16}^{CH}+\dot{E}x_{17}^{CH}-\dot{E}x_{12}^{CH}}{{\left|\dot{E}x\right|}_{Q}^{cd}+\dot{E}x_{12}^{TM}+\dot{E}x_{8}^{TM}-\dot{E}x_{16}^{TM}-\dot{E}x_{9}^{TM}-\dot{E}x_{17}^{TM}}\;$$

Exergy destruction in *HPC* is:

(44) $${\dot{I}}_{HPC}^{}=F_{HPC}^{CH}-P_{HPC}^{CH}\;$$

Exergy destruction can also be calculated based on the Gouy–Stodola theorem:

(45) $${\dot{I}}_{HPC}=T_{0}\cdot {\dot{S}}_{gen}^{HPC}\;$$

in which

(46) $${\dot{S}}_{gen}^{HPC}=\sum^{\;}{\dot{S}}_{e}-\sum^{\;}{\dot{S}}_{i}-\frac{{\dot{Q}}_{cd}}{T_{cd}}={\dot{n}}_{16}\cdot {\bar{s}}_{16}+{\dot{n}}_{9}\cdot {\bar{s}}_{9}+{\dot{n}}_{17}\cdot {\bar{s}}_{17}-{\dot{n}}_{12}\cdot {\bar{s}}_{12}-{\dot{n}}_{8}\cdot {\bar{s}}_{8}+\frac{\left|{\dot{Q}}_{cd}\right|}{T_{cd}}\;$$

The values of the state quantities at the characteristic points are extracted from the results of the ChemSep simulation.

The share of exergy destruction in HPC in the total fuel consumption of the installation is:

(47) $$\psi_{HPC}=\frac{{\dot{I}}_{HPC}}{\left|{\dot{W}}_{1}\right|+\left|{\dot{W}}_{2}\right|+\left|{\dot{W}}_{2}\right|}\;$$

### 3.3. Low Pressure Distillation Column (LPC)

**Figure 8:**
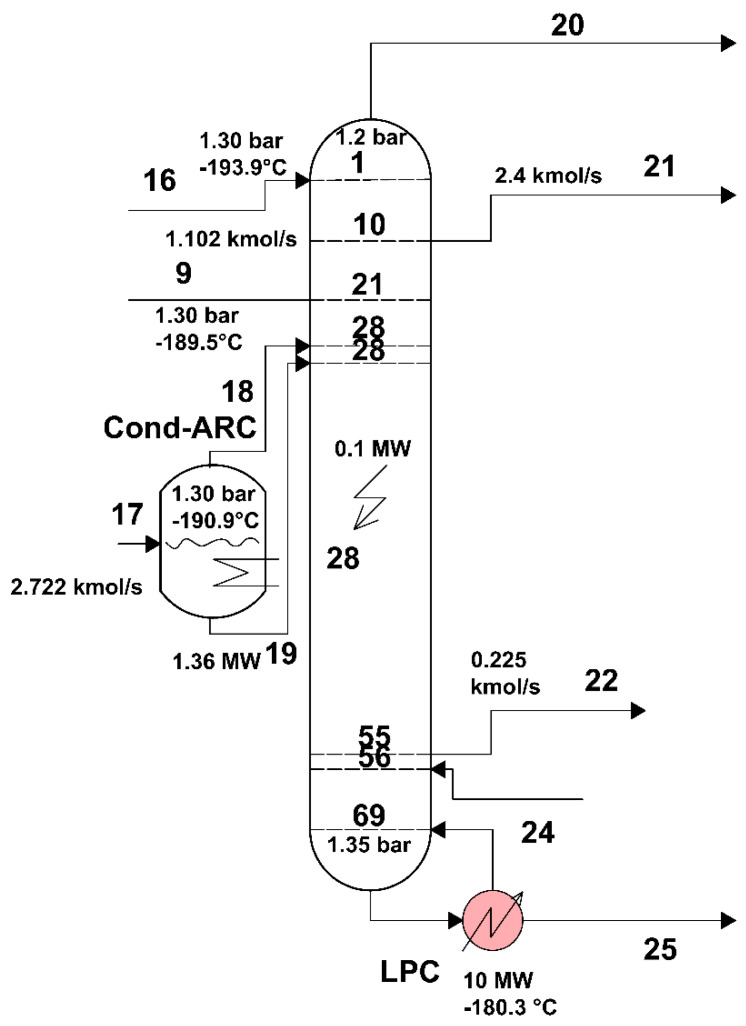
Area of the low-pressure distillation column of the air separation plant.

The area of the low-pressure distillation column, separated from the rest of the installation, is shown in Figure 8.

**Figure 9:**
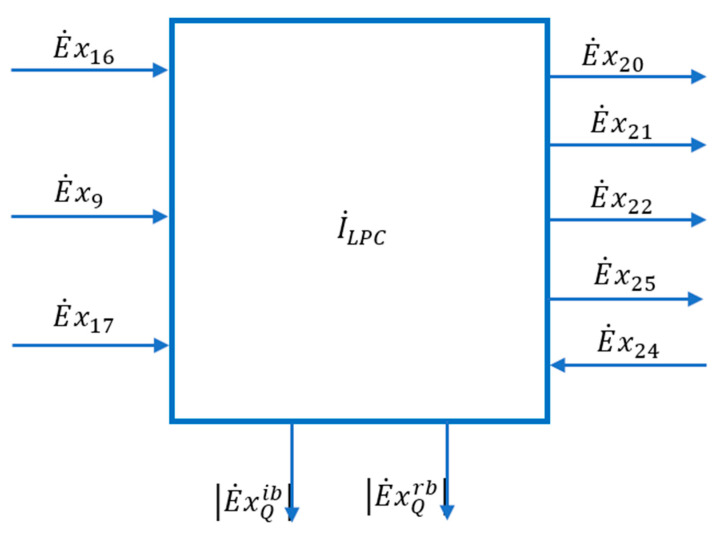
Scheme of interactions with the external environment of the area of the low-pressure distillation column (LPC).

The scheme of the interactions of the LPC area with its exterior can be found in Figure 9.

To determine the senses of the heat exergy exchanges in the reboiler and the intermediate bottle (flash tank), write the general exergetic balance equation in algebraic form (34).

(48) $$\dot{E}x_{Q}^{ib}+\dot{E}x_{Q}^{rb}=\dot{E}x_{20}+\dot{E}x_{21}+\dot{E}x_{22}+\dot{E}x_{25}-\dot{E}x_{16}-\dot{E}x_{9}-\dot{E}x_{17}-\dot{E}x_{24}+\dot{I}\;$$

The heat exergy in the intermediate bottle is:

(49) $$\dot{E}x_{Q}^{ib}={\dot{Q}}_{ib}\left(1-\frac{T_{0}}{T_{ib}}\right)\;$$

It is observed that:

(a) The LPC column receives the heat ${\dot{Q}}_{ib}>0$
(b) Heat is received at a temperature level $\;T_{ib}<T_{0},\left(1-\frac{T_{0}}{T_{ib}}\right)<0$.

In the end,

(50) $$\dot{E}x_{Q}^{ib}<0,\;\dot{E}x_{Q}^{ib}=-\left|\dot{E}x_{Q}^{ib}\right|\;$$

The heat exergy received in the reboiler is calculated as follows:

(51) $$\dot{E}x_{Q}^{rb}={\dot{Q}}_{rb}\left(1-\frac{T_{0}}{T_{rb}}\right)\;$$

It is observed that:

(a) The LPC column receives the heat ${\dot{Q}}_{rb}>0$
(b) Heat is received at a temperature level $T_{rb}<T_{0},\left(1-\frac{T_{0}}{T_{rb}}\right)<0$.

In the end,

(52) $$\dot{E}x_{Q}^{rb}<0,\;\dot{E}x_{Q}^{rb}=-\left|\dot{E}x_{Q}^{rb}\right|\;$$

Replacing and rearranging the terms in Equation (48), we obtain:

(53) $$\dot{E}x_{16}+\dot{E}x_{9}+\dot{E}x_{17}+\dot{E}x_{24}=\dot{E}x_{20}+\dot{E}x_{21}+\dot{E}x_{22}+\dot{E}x_{25}+{\left|\dot{E}x\right|}_{Q}^{ib}+{\left|\dot{E}x\right|}_{Q}^{rb}+\dot{\dot{I}\;}$$

The product of the low-pressure column can be considered as the increase in the chemical exergy of the streams of substance passing through the distillation column and the exergies of the heat offered in the reboiler and the intermediary bottle (flash tank). These heat exchangers are used to extract heat from the high-pressure column (HPC) condensers and the argon column.

Splitting the exergies of the substance currents into the thermo-mechanical and chemical components results in:

(54) $$\dot{E}x_{16}^{TM}+\dot{E}x_{16}^{CH}+\dot{E}x_{9}^{TM}+\dot{E}x_{9}^{CH}+\dot{E}x_{17}^{TM}+\dot{E}x_{17}^{CH}+\dot{E}x_{24}^{TM}+\dot{E}x_{24}^{CH}\;=\dot{E}x_{20}^{TM}+\dot{E}x_{20}^{CH}+\dot{E}x_{21}^{TM}+\dot{E}x_{21}^{CH}+\dot{E}x_{22}^{TM}+\dot{E}x_{22}^{CH}+\dot{E}x_{25}^{TM}+\dot{E}x_{25}^{CH}+{\left|\dot{E}x\right|}_{Q}^{ib}+{\left|\dot{E}x\right|}_{Q}^{rb}$$

The product of the low-pressure column, defined as the increase in chemical exergies of the substances passing through the contour of the low-pressure column, is:

(55) $$P_{LPC}^{CH}=\dot{E}x_{20}^{CH}+\dot{E}x_{21}^{CH}+\dot{E}x_{22}^{CH}+\dot{E}x_{25}^{CH}-\dot{E}x_{16}^{CH}-\dot{E}x_{9}^{CH}-\dot{E}x_{17}^{CH}-\dot{E}x_{24}^{CH}+{\left|\dot{E}x\right|}_{Q}^{ib}+{\left|\dot{E}x\right|}_{Q}^{rb}\;$$

Low pressure column fuel when the product is characterized only by upgrading of chemical exergies is defined by decreasing the thermo-mechanical exergies of the processed currents:

(56) $$F_{LPC}^{CH}=\dot{E}x_{16}^{TM}+\dot{E}x_{9}^{TM}+\dot{E}x_{17}^{TM}+\dot{E}x_{24}^{TM}-\dot{E}x_{20}^{TM}-\dot{E}x_{21}^{TM}-\dot{E}x_{22}^{TM}-\dot{E}x_{25}^{TM}\;$$

Exergy destruction in LPC is:

(57) $${\dot{I}}_{LPC}=F_{LPC}^{CH}-P_{LPC}^{CH}\;$$

It is observed that the value of the exergy destruction deriving from the balance Equation (48) is unique regardless of the way in which the fuel and the product of the column are interpreted.

The different methods of interpreting the product—(a) as the increase in the chemical part of the inlet streams exergies or (b) as the increase in the total exergies—represents the point of view of the researcher in the desire to approach reality.

Estimating the performance as close to reality as possible allows opportunities for the improvement of the system.

The coefficient of exergetic performance of the low-pressure column, depending on the different ways of estimating the fuel and product, is:

(58) $$COP_{LPC}^{CH}=\frac{P_{LPC}^{CH}}{F_{LPC}^{CH}}=1-\frac{{\dot{I}}_{LPC}}{F_{LPC}^{CH}}\;$$

or

(59) $$COP_{LPC}^{TOT}=\frac{P_{LPC}^{TOT}}{F_{LPC}^{TOT}}=1-\frac{{\dot{I}}_{LPC}}{F_{LPC}^{TOT}}\;$$

The destruction of exergy in the low-pressure column can also be calculated with the Gouy-Stodola theorem.

(60) $${\dot{I}}_{LPC}=T_{0}\cdot {\dot{S}}_{gen}^{LPC}\;$$

(61) $${\dot{S}}_{gen}^{LPC}=\sum^{\;}{\dot{S}}_{e}-\sum^{\;}{\dot{S}}_{i}-\sum^{\;}\frac{\dot{Q}}{T}\;$$

(62) $$\begin{array}{c} {\dot{S}}_{gen}^{LPC}={\dot{n}}_{20}\cdot {\overset{ˉ}{s}}_{20}+{\dot{n}}_{21}\cdot {\overset{ˉ}{s}}_{21}+{\dot{n}}_{22}\cdot {\overset{ˉ}{s}}_{22}+{\dot{n}}_{25}\cdot {\overset{ˉ}{s}}_{25}-{\dot{n}}_{16}\cdot {\overset{ˉ}{s}}_{16}-{\dot{n}}_{9}\cdot {\overset{ˉ}{s}}_{9}-{\dot{n}}_{17}\cdot {\overset{ˉ}{s}}_{17}-{\dot{n}}_{24}\cdot {\overset{ˉ}{s}}_{24}\\ -\frac{{\dot{Q}}_{rb}}{T_{rb}}-\frac{{\dot{Q}}_{bi}}{T_{ib}}-\frac{{\dot{Q}}_{iz}}{T_{LPC}} \end{array}$$

### 3.4. Results

The modeling of the operation of the air separation system called upon the ChemSep simulator for the calculation of the separation columns.

The simulation with ChemSep provides, for each main state, the values of flow rates, pressure, temperature, concentration of components O_2_, N_2_ and Ar, values of entropies and molar enthalpies and the phase in which the substance is.

For the other parts of the air separation system that are not involved in a change in the chemical composition of the stream of substances, the calculus is performed based on the EEs program.

#### 3.4.1. Compression Area

The increase in air pressure is achieved in a three-stage compression system with intermediate cooling between stages and final cooling.

To evaluate the performance of the area, attention was focused on exergy losses, with the heat discharged to the outside in the compression system coolers, and on the destruction of exergy due to the irreversibility of compression processes.

The product of the area is the net increase in the total exergy of the air between the inlet and the outlet, and the fuel (resource used) is represented by the electric energy for driving the compressors.

It should be noted that in the conditions of neglecting the consumption of electricity to drive the pumps, the resource used in the compression zone is also the fuel of the global cryogenic air separation installation.

The part of the technological scheme that represents the compression zone is shown in Figure 4.

The values of the total exergies of the inlet and outlet currents in the area as well as of the electrical energies (mechanical compression work) and the exergies of the discharged heat in the cooling system are presented in Table 3.

The exergy values of the input currents and the absolute values of the mechanical works received the plus sign (+) and the values of the exergies of the output currents and the absolute values of the heats transferred to the cooling water were assigned the minus sign (−). In this way, by summing them up, the value of the exergy destruction due to the irreversibility of the compression process was obtained.

If the exergy destructions in each compressor are of interest, they can be calculated with the Gouy–Stodola relation (Equations (29)–(31)). The results are shown in Table 4.

The losses with the exergies of the heat evacuated through the cooling system in the environment are presented for each cooler in Table 5.

The sum of exergy losses and destruction in the compression zone gives:

$${\dot{I}}_{Z,cp}={\dot{I}}_{cp}+L_{Q}=4.958+6.781=11.739\;\mathrm{MW}\;$$

The share of exergy losses and destruction in the compression zone in the fuel consumption of the global installation is:

$$\psi_{cp}=\frac{{\dot{I}}_{Z,cp}}{\left|{\dot{W}}_{1}\right|+\left|{\dot{W}}_{2}\right|+\left|{\dot{W}}_{3}\right|}=\frac{11.739}{31.75}=0.37\;$$

It follows that 37% of the power input of the cryogenic air separation plant is lost or destroyed in the compression zone.

#### 3.4.2. High Pressure Distillation Column

Figure 6 shows the part of the technological scheme of the air separation plant occupied by the high-pressure distillation column (HPC).

The contour of the area also includes the throttling valves and the expander.

The exergetic balance Equation (34) is unique, but the rearrangement of terms from one member of the equation to another is at the discretion of the researcher in his interpretation of the actual product and fuel of the area.

At first glance, the essential role of the column is to separate the components of the mixture, which is related to the increase in the chemical exergy of the output currents compared to those entering the column.

This approach requires the splitting of any total exergy into its thermo-mechanical and chemical parts ($\dot{E}x^{TOT}=\dot{E}x^{TM}+\dot{E}x^{CH})$. A mathematical model solved with EES was used in this purpose.

The computation results are given in Table 6.

The definition of the product of the HPC zone, considering the increase in the chemical component of the currents from inlet to outlet and of the corresponding fuel, according to Equations (41) and (42), and the specification of the corresponding exergetic coefficient of performance Equation (43), leads to the results presented in Table 7.

From the results registered in Table 7, it is observed that after the interpretation that the product of the column is only the increase in the chemical exergy, HPC appears as a technological area with an extremely low exergetic performance, which would lead us to consider another technical solution.

Nevertheless, what really matters is how to manage resources within the area to achieve the desired separation process, or the destruction of exergy within the area does not depend on the interpretation of the product and the zonal fuel (Equation (38)).

If the product and fuel are defined considering the total exergies which, in addition to the chemical part, also consider the pressure and temperature of each stream of substance, elements which count in the devices following the high-pressure column (*HPC*) in the technological flow and for which the product of this column $P_{HPC}\;$ becomes fuel, the results given in Table 8 are obtained.

To calculate the exergy destruction in the HPC, the Gouy–Stodola theorem was used in which the entropy values calculated with ChemSep were used (Equation (45)).

The values of the quantities given by Equation (45) are registered in Table 9.

Entropy transferred from or to the outside associated with heat penetration due to incomplete insulation and heat transferred to the condenser is shown in Table 10.

Entropy generated in the high-pressure column (Equation (46)) and the destruction of associated exergy (Equation (45)) are:

$${\dot{S}}_{gen}^{HPC}=8.861\;\mathrm{kW}$$

$${\dot{I}}_{HPC}=2640\;\mathrm{kW}$$

That leads to a share of exergy destruction in the overall consumption of the installation (Equation (47)) of $\psi_{HPC}=8.3\%$

#### 3.4.3. Low Pressure Distillation Column

Figure 8 shows the part of the technological scheme of the installation occupied by the low-pressure separation column (LPC).

The calculation of the exergy destruction from the LPC was made based on the Gouy–Stodola theorem and the calculation of the column entropy generation (Equation (60)).

The entropy values of the streams of substance in the input and output states of the separation column are calculated with the ChemSep simulator and presented in Table 11.

Multiply the value of the total entropy $\dot{S\;}\left[\frac{kW}{K}\right]$ by (+1) for the output currents and (−1) for the input currents so that the sum results in the entropy flow generated.

Due to the entropy flows transferred from the outside to the reboiler, the intermediate bottle, and due to the penetration of heat through the incomplete insulation, $\frac{{\dot{Q}}_{rb}}{T_{rb}}$, $\frac{{\dot{Q}}_{ib}}{T_{ib}},\;\frac{{\dot{Q}}_{iz}}{T_{LPC}}$ will be multiplied by (−1), thus decreasing Equation (62) from the entropy variation in the system, so that by omitting the entropy transfer, only the entropy generated, as an expression of the exergy destruction, remains.

The destruction of exergy in *LPC* becomes Equation (60):

$${\dot{I}}_{LPC}=734.27\;\mathrm{kW}$$

The share of *LPC* exergy destruction in the overall electricity consumption of the installation is:

$$\psi_{LPC}=\frac{{\dot{I}}_{LPC}}{\left|{\dot{W}}_{1}\right|+\left|{\dot{W}}_{2}\right|+\left|{\dot{W}}_{3}\right|}100=\frac{734.27}{31750}100=2.3\%$$

## 4. Discussion

The energetic analysis accounts only for the losses of energy thrown out into the environment of the system. The energetic analysis does not account for the quality of the lost energy.

Only the exergetic analysis that couples the first and second principles of thermodynamics reveals not only losses but also destructions (consumption) of useful energy, thereby making the correlation between the intensive parameters of the system and those of the environment.

In cryogenic systems analysis, the use of exergetic analysis is almost compulsory, because the lowest is the operating temperature and the highest is the entropy generation due to an irreversibility.

The splitting of the total exergy of each current of substance into its physical and chemical components makes it possible to define, in different ways, the product and the fuel of every functional zone and, finally, the local COP. In this way, more alternatives of discussion are found, enriching the knowledge about the operation of the system.

The analysis found that the most inefficient part of the ASU is the compression area.

The loss and the destruction of the compression area in relation to the fuel of the entire plant is 37%.

The second destructive area is represented by the separation columns [17,18]. The local coefficient of performance of the HPC is 62.66%. The exergy destruction in the local fuel of the HPC is 37.34%, but when calculated as a share of the total input of the entire plant, is only 8.3%.

The results are comparable to other studies [19,20,21,22,23,24,25]. All studies performed on air separation units with cryogenic distillation columns report that about one third of the global system’s power input is consumed in the compression stage. As in the present work, Cornelissen and Hirs [21] and Spali et al. [25] reported much less exergy destruction in the distillation column compared to the compression unit.

In the present work, the model of exergy analysis refers to specific boundaries of the compression unit (Figure 4) and of the high-pressure (Figure 6) and low-pressure (Figure 8) distillation columns; the throttling valves and the condenser-reboiler connecting the two distillation columns is not included. This last heat exchanger introduces significant destruction due to heat transfer at a finite difference of temperature

## 5. Conclusions

This paper presents a model of a detalied exergetic analysis of some key parts of a cryogenic air separation unit, a model that could be integrated into a functional or design optimization procedure.

The exergetic method is particularly productive in low temperature operating systems where any irreversibility due to the low temperature level leads to a large generation of entropy and, consequently, a large exergy consumption (destruction).

Exergy analysis reveals the real measure of losses and exergy destruction, enabling the discovery of methods for waste energy recovery or better investments for lowering the inputs.

The exergy analysis discusses the different ways in which the coefficient of performance of a key part of the system could be viewed in the aim of getting close to reality.

The paper points out that, for the analyzed installation, the compression unit is by far the most destructive part, suggesting in this way the use of better compressors and the recovery of the heat transferred to the compressors’ cooling system.

Further analysis should be developed on the recuperative internal heat exchanger that plays, at its turn, a leading part in the economy of the system.

## Figures and Tables

**Table 1:** Compressors input powers.

Compressor	$\left|\dot{W}\right|\left[kW\right]$
1	10,292
2	10,712
3	10,751
Global fuel of the installation F	31,755

**Table 2:** Values of the exergetic products.

Substance	$\dot{E}x^{TM}\left[kW\right]$	$\dot{E}x^{CH}\left[kW\right]$	$\dot{E}x^{TOT}\left[kW\right]$
${\mathrm{GO}}_{2}$	377.4	2752	3129.4
${\mathrm{GN}}_{2}$	279	835.8	1114.8
Lar	151.5	89.53	241
Global installation product P			4485.2

**Table 3:** The values of the total exergies of the air currents, of the driving power of the compressors and of the exergies of the heat evacuated by the cooling system.

Exergy Current	Exergy [MW]
AIR	0
$\left|{\dot{W}}_{1}\right|$	10.29
$\left|{\dot{W}}_{2}\right|$	10.71
$\left|{\dot{W}}_{3}\right|$	10.75
$\left|\dot{E}x_{Q_{1}}\right|+{\dot{I}}_{\Delta p_{1}}$	−2.3
$\left|\dot{E}x_{Q_{2}}\right|+{\dot{I}}_{\Delta p_{2}}$	−2.32
$\left|\dot{E}x_{Q_{3}}\right|+{\dot{I}}_{\Delta p_{3}}$	−2.161
$\dot{E}x_{6}^{TOT}$	−20.015
${\dot{I}}_{cp}=\sum \dot{E}x_{i}-\sum \dot{E}x_{e}$	4.954

**Table 4:** Exergy destruction in the compression stages.

Compression Stage	$\mathrm{Destruction}\;\mathrm{of}\;\mathrm{Exergy}\;{\dot{I}}_{cp}$ [MW]
1	1.659
2	1.647
3	1.652
$\mathrm{Total}\;{\dot{I}}_{cp}$	4.958

**Table 5:** Losses with heat exergies evacuated in the coolers of the compression stages.

Cooler	$\mathrm{Exergy}\;\mathrm{Heat}\;\mathrm{Loss}\;\left|\dot{E}x_{Q}\right|+{\dot{I}}_{\Delta p}\;\left[MW\right]$
1	2.3
2	2.32
3	2.161
$\mathrm{Total}\;L_{Q}$	6.781

**Table 6:** Values of thermo-mechanical and chemical exergies of the flows entering and leaving the control volume of the HPC (Figure 6 and Figure 7).

The Current	$\dot{E}x^{TM}$ [MW]	$\dot{E}x^{CH}\left[MW\right]$
12	31.409	0
8	5.366	0
$\left|\dot{E}x_{Q}^{cd}\right|$	31.622	
16	23.132	0.674
9	3.690	0
7	15.222	0.142

**Table 7:** Values of the product, fuel and exergetic coefficient of performance of the HPC considering that its purpose is to increase the chemical component of the currents by separation.

$P_{HPC}^{CH}$ [MW]	$F_{HPC}^{CH}$ [MW]	$COP_{ex\_HPC}^{CH}\%$
0.816	26.353	3

**Table 8:** Values for product, fuel and coefficient of performance when it is considered that the purpose of HPC is to increase the total exergy by separating the streams of substance.

$P_{HPC}^{TOT}\left[MW\right]\;$	$F_{HPC}^{TOT}\left[MW\right]\;$	$COP_{ex\_HPC}^{TOT}\%$
42.86	68.397	62.66

**Table 9:** Entropy values and molar flow rates of the substances at the boundary separating the HPC from the outside.

Condition	$\bar{s}\left[\frac{kJ}{kmol\;K}\right]\;$	$\left[\dot{n}\right]\frac{kmol}{s}\;$	$\dot{S}\left[\frac{kW}{K}\right]\;$
8	−41.919	0.675	−28.3
9	−39.4896	0.675	−26.66
12	−55.4484	3.825	−212.1
16	−96.496	1.102	−106.4
17	−93.125	2.723	−253.6

**Table 10:** Entropy flux values transferred between HPC and outside.

Device	$\mathrm{Transferred}\;\mathrm{Entropy}\;\frac{\dot{Q}}{T}\left[\frac{kW}{K}\right]$
Condenser	−156.1
Heat penetration from the outside	1.048

**Table 11:** Entropy and molar flow rates values at the boundary separating the LPC from the outside.

Condition	$\bar{s}\left[\frac{kJ}{kmol\;K}\right]\;$	$\left[\dot{n}\right]\frac{kmol}{s}\;$	$\dot{S}\left[\frac{kW}{K}\right]\;$
9	−39.4896	0.765	−26.66
16	−96.496	1.102	−106.4
17	−93.125	2.723	−253.6
20	−40.08	1.367	−55.78
21	−36.82	2.4	−88.37
22	−32.247	0.225	−7.256
24	−105.262	0.2173	22.87
25	−107.998	0.7251	−78.31
$\frac{{\dot{Q}}_{rb}}{T_{rb}}$			−160.7
$\frac{{\dot{Q}}_{bi}}{T_{bi}}$			−16.59
$\frac{{\dot{Q}}_{iz}}{T_{LPC}}$			−1.079
${\dot{S}}_{gen}$	2.464 [kW]		

## Data Availability

Not applicable.

## Nomenclature

ASU	air separation unit
Cp	compressor
${\dot{E}}_{el}$	current of electrical energy (exergy), kW
$\dot{E}x$	current of exergy, kW
F	exergetic fuel, kW
$GN_{2}$	current of gaseous Nitrogen, kmol/s
$GO_{2}$	current of gaseous oxygen, kmol/s
HPC	high-pressure column of distillation
LPC	low-pressure column of distillation
LAr	current of liquid Argon, kmol/s
$\dot{Q}$	current of heat, kW
P	exergetic product, kW
$\dot{W}$	mechanical power, kW
$\dot{E}x^{TM}$	current of thermo-mechanical exergy, kW
$\dot{E}x^{CH}$	current of chemical exergy, kW
$\bar{h}$	molar enthalpy, kJ/kmol
$\dot{I}$	current of exergy destruction due to internal irreversibility, kW
L	current of exergy loss, kW
n	current of substance, kmol/s
p	pressure, Pa
$\bar{R}$	universal constant of gases, J/kmol/K
$\bar{s}$	molar entropy, kJ/kmol
T	temperature, K
$\bar{x}$	molar composition
Subscripts	
0	environment, in equilibrium with the environment
Cd	condenser
cp	compressor
e	exit
ex	exergetic
HPC	high-pressure column
i	inlet
iz	insulation
L	loss
LPC	low-pressure column
rb	reboiler
Q	heat
Superscript	
Cd	condenser
TOT	total
CH	chemical
ib	intermediary bottle
rb	reboiler
TM	thermo-mechanical
Greek Symbol	
Ψ	share of an exergetic loss or destruction in the fuel consumption
